# Functional evaluation of a novel kisspeptin analogue on the reproduction of female goldfish

**DOI:** 10.1038/s41598-022-25950-4

**Published:** 2022-12-19

**Authors:** Hanieh Rabouti, S. Mohsen Asghari, Reihaneh Sariri, Saeed Balalaie, AbdolMajid Valipour, Navid Omidian, Behrooz Heidari

**Affiliations:** 1 Department of Biology, Faculty of Sciences, University of Guilan, Rasht, Iran; 2 Institute of Biochemistry and Biophysics (IBB), University of Tehran, Tehran, Iran; 3 Peptide Chemistry Research Center, K.N. Toosi University of Technology, Tehran, Iran; 4 Department of Marine Sciences, Caspian Sea Basin Research Center, University of Guilan, Rasht, Iran

**Keywords:** Biochemistry, Biotechnology, Physiology

## Abstract

Kisspeptin (kp) is a key regulator of reproduction, which stimulates sexual maturation and gametogenesis in mammals, amphibians, and teleosts. In the present study, to enhance the biological activity of kp10, a novel analog (referred to as M-kp10) was designed based on the endogenous goldfish variant, in which phenylalanine 6 was substituted by tryptophan and the N-terminus was acetylated. Compared with the native kp-10 and salmon gonadotropin-releasing hormone (GnRH3), the effect of M-kp10 on sexual hormones and reproductive indices as well as the expression of *kiss1*, *cyp19a1*, and *kiss1ra* genes in goldfish (*Carassius auratus*) was investigated. In practice, peptides were synthesized based on the standard Fmoc-solid-phase peptide synthesis and purified by employing RP-HPLC, followed by approving their structure using ESI-MS. The results showed that M-kp10 increased significantly 17,20β-DHP, LH, FSH and E2 as well as fecundity, hatching and fertilization percentages than the other peptides. Histological studies revealed that M-kp10 led to the faster growth of ovarian follicles compared to the kp-10 and GnRH3. The genes of *cyp19a1*, *kiss1ra*, and *kiss1* were remarkably more expressed after treatment with M-kp10. In conclusion, the results indicated the superiority of M-kp10 over kp-10 in inducing sexual maturation and accelerating the percentage of fecundity, suggesting that M-kp10 could be a promising candidate for application in the artificial breeding of fish.

## Introduction

The reproductive cycle of the fish includes gamete development, maturation, and spawning, which initiates by the gonadotropin-releasing hormone (GnRH). GnRH affects gonadotropic cells in the pituitary gland and stimulates the production of gonadotropins^1,2^ such as LH and FSH, leading to follicular growth^3^.

Kisspeptin (Kp) is a regulator of reproduction, which stimulates sexual maturation, gametogenesis and ovulation through hypothalamic-pituitary-gonadal (HPG) axis in human, mammals, amphibians and teleosts^4–7^. Apart from hypothalamus, kisspeptin system was also found in extrahypothalamic tissues including limbic and paralimbic brain regions, placenta, pancreas, ovary and liver in peripheral areas^8,9^. However, most data indicate that teleost Kiss neurons are not the principal regulators of GnRH and LHRH, as in the case for model mammalian species^10^.

Kisspeptins are a family of structurally related peptides, encoded by the *KISS1/Kiss1* gene, that act through binding and subsequent activation of the G protein-coupled receptor GPR54, which also known as the kisspeptin receptor (Kiss1R). The Kiss1 gene product is translated into a 145 amino acids residue precursor that is further cleaved into a 54-residue peptide, originally called metastin or Kp-54^11^. Additional cleavage of metastin results in the production of shorter peptides, including Kp-16, Kp-14, Kp-13, and Kp-10 based on their length^11^.

In most teleosts, the kisspeptin system comprises two types of kiss genes, Kiss1 and Kiss2, and two kisspeptin receptors, Kissr1 and Kissr2. Administration of Kiss1 and Kiss2 does not exert similar effects in different teleosts. In some cases, e.g. goldfish (*Carassius auratus*)^1^, Kiss1 stimulates the secretion of LHRH much more effectively than Kiss2, whereas the opposite effects were observed in other species such as European sea bass (*Dicentrarchus labrax*) and orange-spotted grouper (*Epinephelus coioides*)^12^.

Numerous kisspeptin analogs have been synthesized in structure–activity studies^13^ Based on D-amino acid scanning analysis of human kp-10, the five C-terminal residues, including ^6^Phe-Gly-Leu-Arg-Tyr^12^ (corresponding to ^6^Phe-Gly-Leu-Arg-Phe^10^ in teleosts) are stereochemically of high importance for proper kisspeptin receptor activation. Among these residues substitution of either Phe6, Arg9, or Phe10 showed the highest impact on the agonistic activity^14–16^. Importantly, these three residues lie on one face of a helix and define a pharmacophore site for kisspeptin^14^. Structure–activity studies at the Phe10 resulted in an improved activity by substitution with Trp^14,17^. With the same rationale and considering the role of Phe6 in the bioactivity of kp-10, we aim to substitute Phe6 with Trp to enhance its bioactivity. In addition, the N-terminus of the peptide is acetylated. This modification was due to the fact that N-terminal acetylation was found to enhance the relative resistance to proteolytic degradation and circulation half-life in different peptides^18^. The novel kp-10 analogue containing Phe6Trp substitution and the N-terminal acetylation referred to as modified kp-10 (M-kp10). We exploited goldfish (*Carassius auratus*) as a laboratory model^19^ to assess the bioactivity of M-kp10 in vivo, and native kp-10 and synthetic GnRH3 was compared with the novel peptide.

## Results

### Hormonal analysis

**Figure 1:**
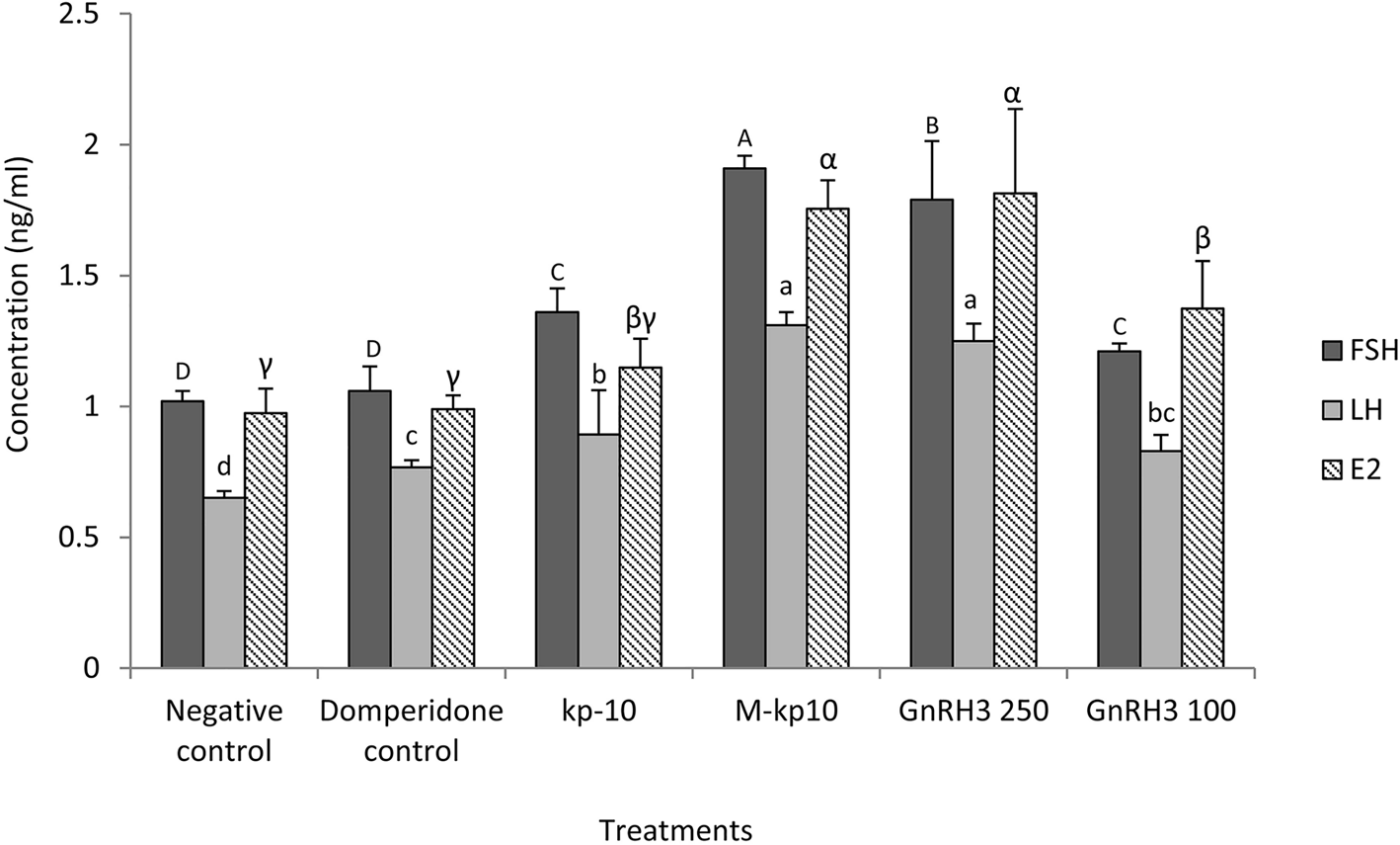
Variations of the luteinizing hormone (LH), the follicle stimulating hormone (FSH) and the 17β estradiol (E2) in the female goldfish treated with kp-10, M-kp10 and GnRH3, 6 h post-injection. The different letters above columns are showing significant differences based on one-way ANOVA and Tukey’s post hoc test.

The various hormones including 11KT, 17,20β-DHP, cortisol, E2, LH, FSH, and LPL were analyzed after injecting the peptides M-kp10, kp-10 and GnRH3s. The level of E2 was significantly higher in the M-kp10 and GnRH3 (250 µg/kg) injected groups (*p* = 0.001, F = 16.64, df = 5) (Fig. 1). It was observed that, compared to the kp-10 and GnRH3 (100 µg/kg) groups, FSH (*p* = 0.001, F = 36.18, df = 5) was maximized after receiving M-kp10 (Fig. 1). The maximum level of LH (*p* = 0.002, F = 31.32, df = 5) was recorded in M-kp10 and GnRH3 (250 µg/kg). The maximum level of FSH was observed in M-kp10.

**Figure 2:**
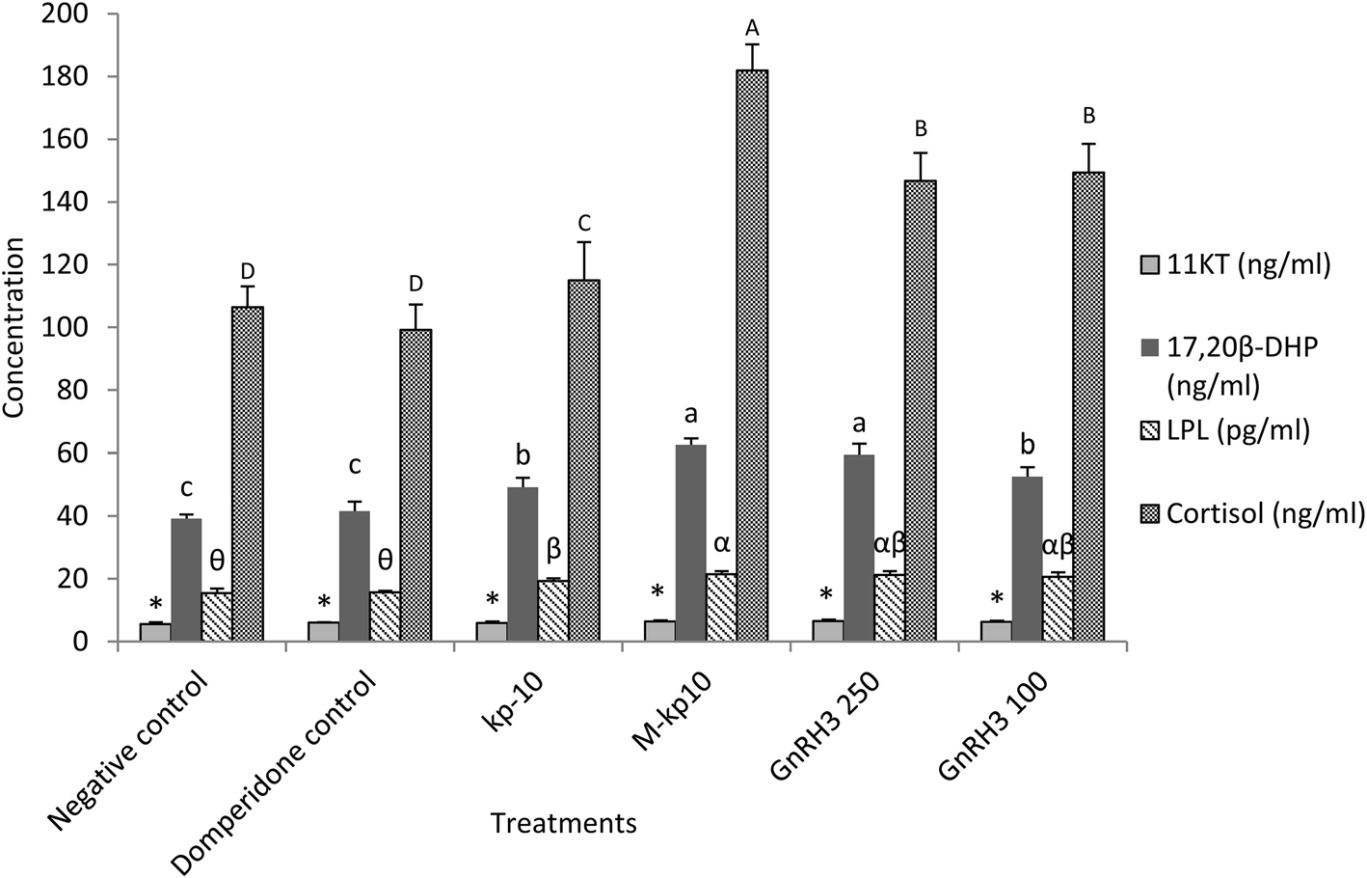
Variations of 17α-20β-Hydroxy-4-peregnen-3-one (DHP), 11-keto testosterone (11KT), lipoprotein lipase (LPL) and cortisol in the female goldfish treated with kp-10, M-kp10 and GnRH3, 6 h post-injection. The different letters above columns are showing significant differences based on one-way ANOVA and Tukey’s post hoc test.

There were no remarkable changes in 11KT in different groups (*p* = 0.852, F = 98.89, df = 5) and the 17,20β-DHP level was significantly higher in the M-kp10 and GnRH3 (250 µg/kg) compared to kp-10 (*p* = 0.001, F = 22.43, df = 5) (Fig. 2). Moreover, the cortisol amounts increased significantly in all treatments (*p* = 0.001, F = 37.63, df = 5) (Fig. 2). All treatments led to increased LPL levels in the goldfish compared to the controls (*p* = 0.001, F = 20.02, df = 5) (Fig. 2).

### Expression levels of *kiss1*, *cyp19a1* and *kiss1ra* genes

**Figure 3:**
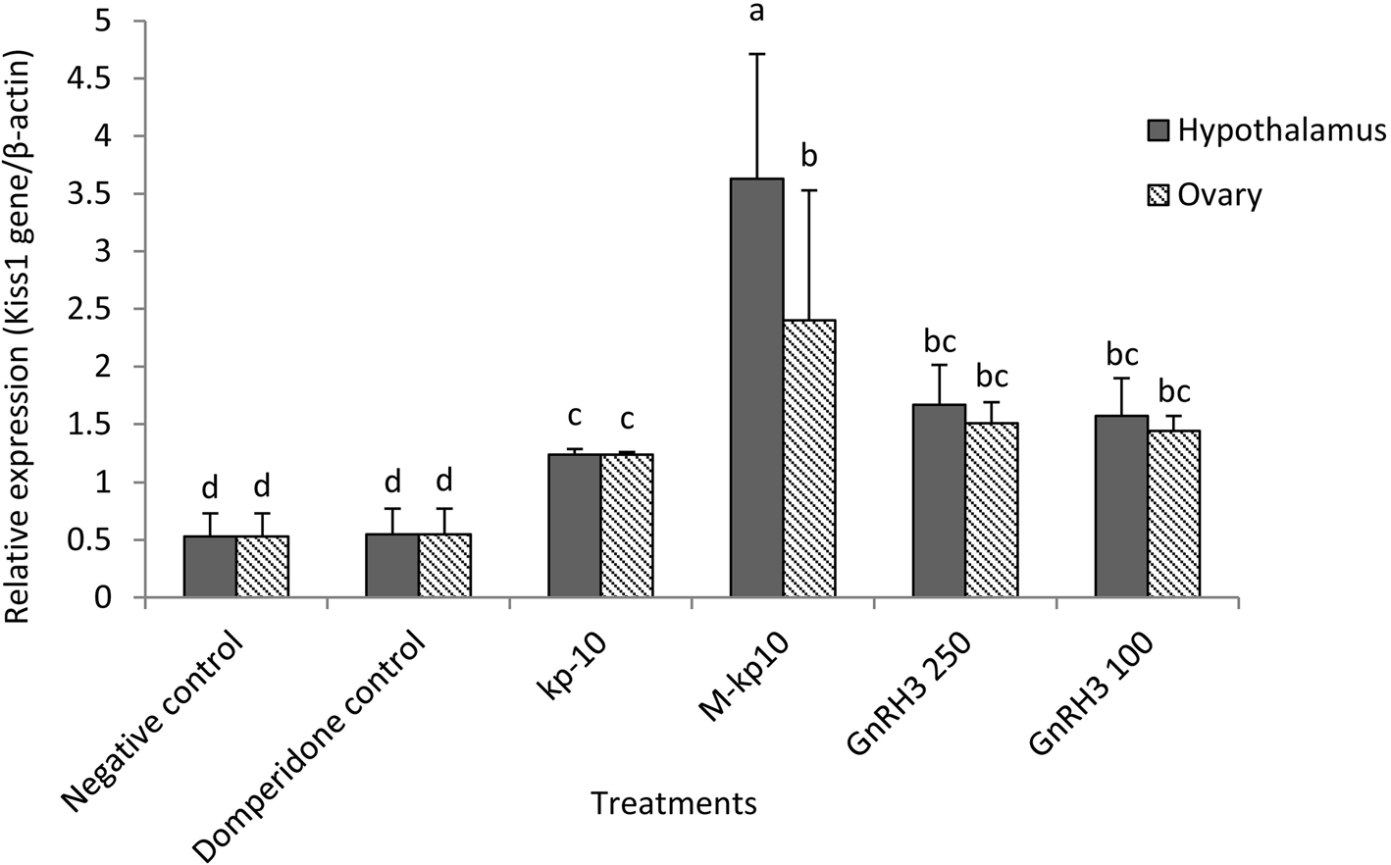
Changes in the expressions of *kiss1* gene in the ovarian and hypothalamus tissues of female goldfish based on the RT-PCR (The *actb* is considered as the control). The different letters above columns are showing significant differences based on one-way ANOVA and Tukey’s post hoc test.

**Figure 4:**
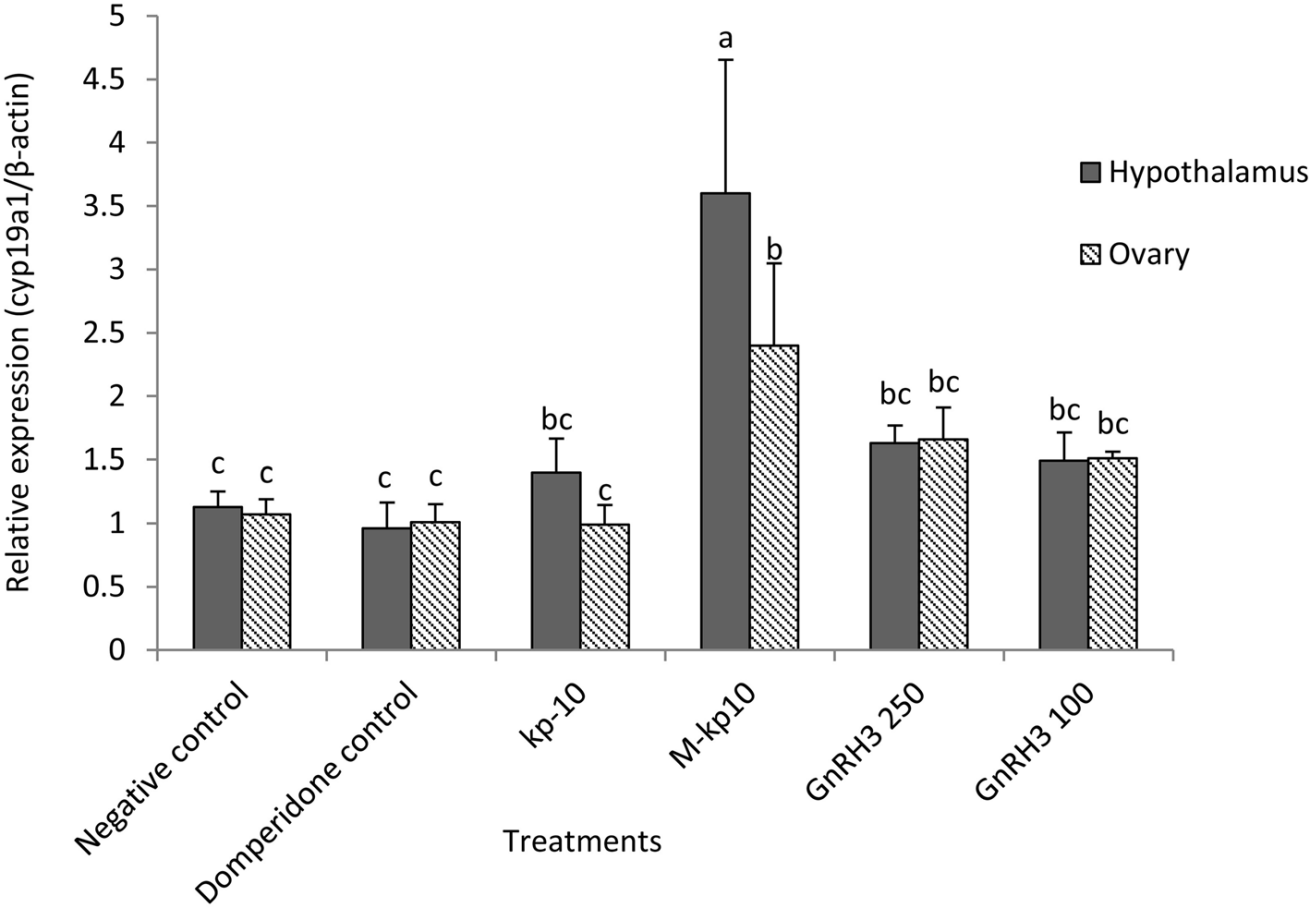
Changes in the expressions of *cyp19a1* gene in the ovarian and hypothalamus tissues of female goldfish based on the RT-PCR (The *actb* is considered as the control). The different letters above columns are showing significant differences based on one-way ANOVA and Tukey’s post hoc test.

**Figure 5:**
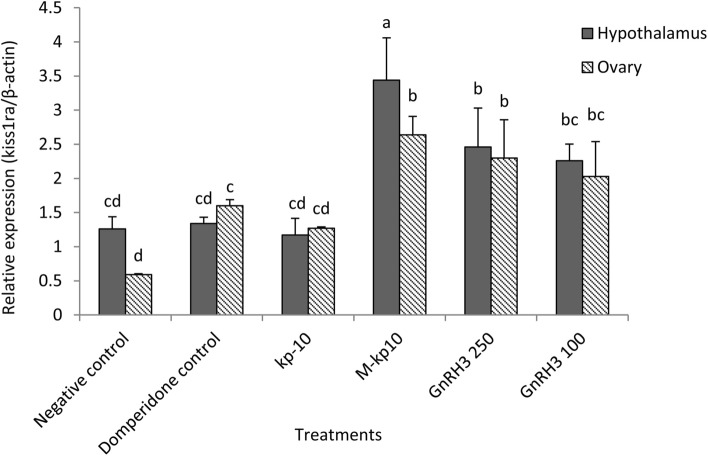
Changes in the expressions of *kiss1ra* gene in the ovarian and hypothalamus tissues of female goldfish based on the RT-PCR (The *actb* is considered as the control). The different letters above columns are showing significant differences based on one-way ANOVA and Tukey’s post hoc test.

All the qPCR assays described in this approach had high efficiencies (> 95% ± 0.82). The expression level of *kiss1*, *kiss1ra*, and *cyp19a1* genes were assessed in the ovarian and hypothalamic tissues received M-kp10, kp-10, and GnRH3. There was a significant correlation coefficient between the relative expression of the *kiss1* (*p* < 0.01: r = 78.44), *kiss1ra* (*p* < 0.01: r = 70.34) and *cyp19a1* genes (*p* < 0.01: r = 64.25) in the hypothalamic and ovarian tissues. The highest expression of these genes was recorded in hypothalamic tissue in M-kp10 treatment (*p* = 0.001). As indicated in Figs. 3, 4, and 5, there were significant changes in the expression levels of *kiss1* (*p* = 0.003, F = 9.26, df = 11), *kiss1ra* (*p* = 0.002, F = 7.82, df = 11)*,* and *cyp19a1* (*p* = 0.002, F = 9.57, df = 11) by injecting kisspeptins and GnRH3. M-kp10 led to a much higher rise in hypothalamic *kiss1* (Fig. 3), *cyp19a1* (Fig. 4) and, *kiss1ra* (Fig. 5) mRNA levels than kp-10 compared to the control group (*p* = 0.001). Further, compared to M-kp10, the variations of hypothalamic *kiss1*, *kiss1ra*, and *cyp19a1* mRNA levels were less significant after GnRH3 treatments. In the ovarian tissue, M-kp10 resulted in improving the expression levels of *kiss1*, *kiss1ra*, and *cyp19a1* genes significantly, while fewer changes were observed following kp-10 and GnRH3 treatments.

### Ovarian histology

**Figure 6:**
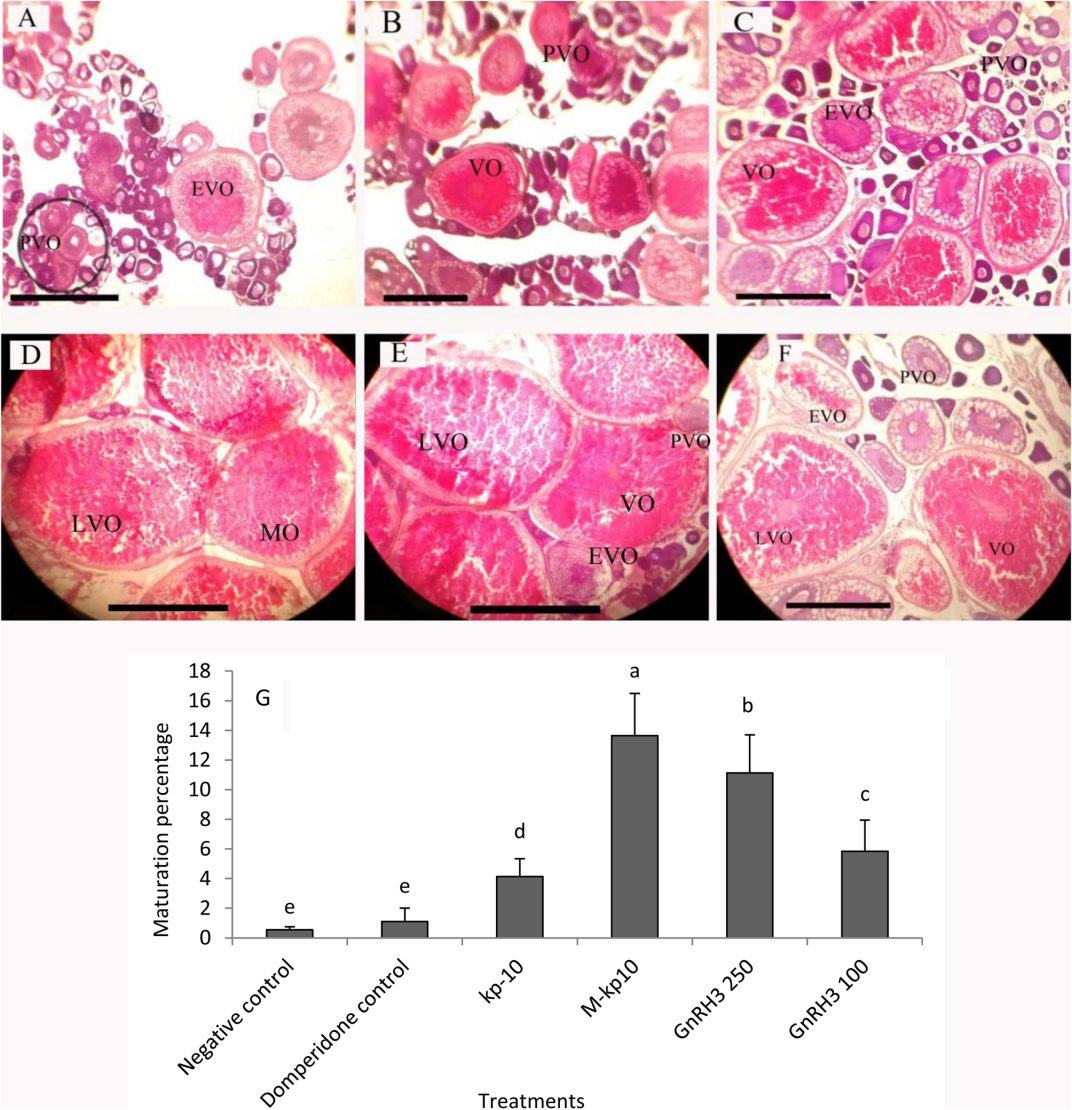
Photomicrographs of H&E-stained ovary in the female goldfish. (**A**) Negative control, (**B**) Domperidone control, (**C**) kp-10, (**D**) M-kp10, (**E**) GnRH3 (250 μg/ml), and (**F**) GnRH3 (100 μg/ml) and maturation percentage of oocytes (**G**). Abbreviations: PVO, pre-vitellogenic oocyte; EVO, early-vitellogenic oocyte; VO, vitellogenic oocyte; LVO, late-vitellogenic oocyte; PMO, pre-mature oocyte; MO, mature oocyte. Scale bar = 500 µm.

Histological structure of ovary exhibited the generalized bony fish with ovary structure during 24 h post-injection (Fig. 6A–F). Based on the microscopical examination of the female gonads of *C. auratus*, most oocytes were detected at the pre-vitellogenic stage (PVO) in the (A) negative control and domperidone Control (B). The oocyte number in the vitellogenic stage (VO) increased following kp-10 treatment (C), in which the yolk-filled sacs were observed between the cortical alveoli in the peripheral cytoplasm. In addition, the ovary became more developed and reached the final maturation stage (ripe oocytes) after (D) M-kp10 and (E) GnRH3 (250 μg/ml) treatments. The results reflected a greater percentage of mature oocytes in the M-kp10-treated group than in the kp-10 and GnRH3-injected ones (*p* = 0.001, F = 28.75, df = 5) (Fig. 6G). Based on statistical analysis, mature oocytes increased in the M-kp10 group compared to the others (Fig. 6G) (*p* = 0.001).

### Fecundity and percentage of fertilization and hatching

**Figure 7:**
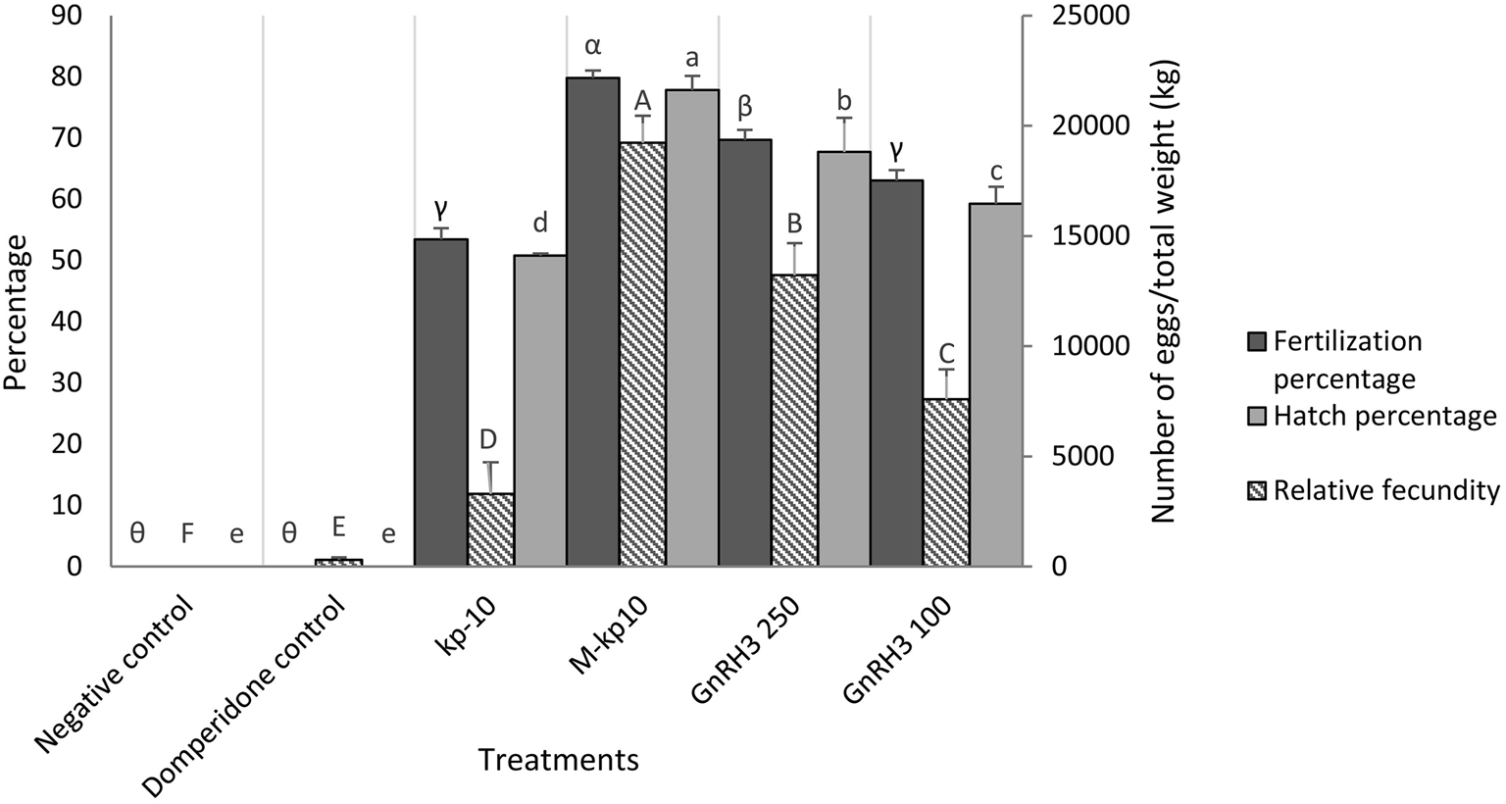
Relative fecundity, percentage of fertilization and hatching in the female goldfish treated with kp-10, M-kp10 and GnRH3. The different letters above columns are showing significant differences based on one-way ANOVA and Tukey’s post hoc test.

Injection of kp-10, M-kp10, and GnRH3 (100 µg/kg) led to significant differences in the relative fecundity (*p* = 0.001, F = 598.81, df = 5), percentage of fertilization (*p* = 0.001, F = 3350.75, df = 5), and hatching (*p* = 0.001, F = 2154.01, df = 5) (Fig. 7). The highest fecundity was recorded in fish receiving M-kp10. Increasing in GnRH3 concentration from 100 to 250 µg/kg improved fecundity, fertilization, and hatching rates but not as much as the M-kp10 (*p* = 0.001).

## Discussion

The members of the HPG axis are broadly used for accelerating and synchronizing oocyte maturation in the fishery industries. Given that milt amount is not considered a limiting factor in the artificial reproduction and most hormonal manipulations are utilized to increase the spawning of female fish, we designed, synthesized, and characterized a new kp-10 analog (M-kp10) to increase reproductive efficiency in female fish.

Numerous kisspeptin analogs have been synthesized to improve their biological activity and/or stability, including those containing substitutions with unnatural amino acids and/or chemical modifications^13,20^. Substitution of Gly-Leu dipeptide bond, which is located at the C-terminal moiety of Kiss1 and is susceptible to proteolytic degradation with metalloproteases, improved its stability against proteolytic degradation^21^. In addition, Asami et al. reported that substitution of Arg9 improved the bioactivity compared to kp-10 and cleavage resistance^22^. The strategy of stabilizing kp-10 by N-terminal modification was indicated in kp-10 analogue C6 in which an albumin binding motif inserted in the isoGlutamyl on the N-terminal amine, arginine ω-methylated at position 9, and triazole inserted between the leucine and the glycine. Moreover, C6 analogue was more active than kp-10 in ewes^23,24^. FTM080, a kisspeptin receptor agonist, indicated an increased half-life in murine serum, but lower activity than native kp-10 in ewes^25^. An interesting analogue that indicated improved serum stability was compound 26 (C26) which designed by N-terminal truncation of kp-10^26^. TAK-448 and TAK 683 are two kp-10 analogues with nine residues that exhibited comparable Kiss1 receptor-binding affinity and potency and increased half-life than kp-10 in vivo^27^. In current research, based on in vivo studies in goldfish, including analyses of sexual hormones, reproductive indices and the expression levels of *kiss1*, *cyp19a1*, and *kiss1ra* genes, M-kp10 promoted sexual maturation and gametogenesis more efficiently than the native kp-10.

Orsini et al. reported a rise in the activity of kp-10 following substitution of Phe with Trp at C-terminus^14^. Moreover, substitution of Phe6, Arg9 and Phe10 with Ala abolished the agonistic activity of kp-10^14–16^. As a results, Phe6 and Phe9 were proposed as critical residues for binding to the hydrophobic pocket of the receptor^14^. Likewise, Gutierrez-Pascual et al. outlined the critical role of Phe6 in the agonistic activity of rat kp-10^15^. The results of current study that substitution of Phe6 with Trp improve the bioactivity of kp-10, along with previous studies underscores the important role of Phe6 in the activity of kp-10.

The limiting circulation half-life of kp-10 is an important obstacle for its application. The shorter forms of kisspeptin are less potent than the longer ones when administered peripherally due to a smaller circulation half-life^28^. Due to the limitations of production and improvement of the larger molecules, the chemical modification of the shorter molecule kp-10 is an alternative strategy to enhance the stability and activity. We speculate that the improved activity of M-kp10 can be attributable to the N-terminal acetylation, as it was shown to prevent degradation and increases their half-life in circulation^18,29^. In the present study, both strategies, i.e. amino acid replacement and chemical modification were utilized to promote the activity of kp-10 in *C. auratus*.

To compare the activity of kp-10, M-kp10, and synthetic GnRH3, we examined the reproductive indices, hormones level in plasma (LH, FSH, 11KT, 17-20β-DHP, E2, cortisol, and LPL), and ovarian histology as well as the expression levels of *kiss1*, *kiss1ra,* and *cyp19a1* genes from the hypothalamic and ovarian tissues. The highest FSH and LH levels were observed in the M-kp10-injected group. Valipour et al. proposed that kisspeptin can play a role in secreting gonadotropins such as LH and FSH^30^. In bony fish, LH, FSH, progesterone, estradiol, and testosterone hormones stimulate oocyte growth and maturation and control these functions^31^. Whereas Li et al. reported that kp-10 cannot stimulate the secretion of LH in primary pituitary cells, current study in agreement with Somoza and colleagues suggests a direct pituitary action on LH secretion^1,10^.

E2 should be increased while enhancing FSH and 17-20β-DHP should follow while raising LH^32^. In this study increase in LH and FSH due to the injection of M-kp10 and GnRH3 (250 µg/kg) has caused an increase in E2 and 17-20β-DHP secretion.

Given that 17-20β-DHP is the main maturing hormone in fish, M-kp10 plays an important role in the final maturation of oocytes^28^. Tokumoto et al. reported that prolonged incubation with 17-20β-DHP in vivo can lead to ovulation, which reflects the role of 17-20β-DHP in oocyte maturation in freshwater fish^33^. In this study, the highest amount of 17-20β-DHP was observed in the M-kp10 and GnRH3 (250 µg/kg) groups, and also the highest percentage of oocyte maturation was observed in these two groups.

Injection of two synthetic kisspeptins into scombroid fish showed significant increases in E2 levels^34^. Significant increases in E2 levels were observed in male and female Nile tilapia exposed to the kisspeptin-10^35^. Significant increase in the levels of the E2 were observed in Chub mackerel (*Scomber japonicus*) that were affected by kisspeptin-15^34^. In the present study, E2 levels in M-kp10 and GnRH3 (250 µg/kg) treatments showed a significant increase compared to other groups.

In the present study, the highest amount of cortisol was related to M-kp10 treatment. Further, 17-20β-DHP is involved in both the hydration and final maturation of the oocyte, while cortisol is only involved in its hydration in vitro^36^. Suzuki et al. found that a rise in cortisol during spawning catfish (*loricariid catfishes*) may be attributed to fish physiological activities like osmotic regulation and energy supply processes which occur at the same time as fish reproduction^37^. In this study, cortisol levels were significantly higher in the M-kp10 group compared to all other groups. Milla et al. showed that hydration of oocytes can be induced in vitro by cortisol and these data probably explain the high level of fecundity in the M-kp10 group^36^.

None of the treatments showed an increase in 11KT concentration. Plasma LPL activity increases during oocyte maturation and reaches its maximum after vitellogenesis^38^. In the present study, the treatments significantly increased the LPL activity compared to the control samples. The highest level of LPL activity in the M-kp10 group can indicate oocyte maturation and confirm the effectiveness of M-kp10.

The various effects of kisspeptin treatments on the hormones can be ascribed to the independent function of this neuropeptide in different tissues. This means that brain kisspeptin can be synthesized independently of that in ovaries and other tissues and exert its physiological role. Kisspeptin plays different physiological roles in the hypothalamic and ovarian tissues. Furthermore, the role of kisspeptins can be related to different times. The results of the previous studies have indicated that kiss1 mRNA is expressed in the fish brain and can participate in reproduction, feeding, and behavior^39^. Kiss1 has been reported in the theca and granulosa cells of the ovarian follicles in catfish^39^. According to Chang et al., kisspeptin directly affects pituitary hormone secretion in some mammals and goldfish^40^.

Expression of the *kiss1* and *kiss1ra* genes has also been reported in brain neurons^41^ as well as in organs such as the testes, ovaries, pituitary, and pancreas^42^. Kisspeptin increases the secretion of gonadotropins, but for this purpose, it must first stimulate GnRH-producing neurons in the hypothalamus, which must increase Kiss1 receptors on their cell membrane to be stimulated^43,44^. Therefore, the action of kisspeptin requires the expression and presence of *kiss1ra* in GnRH-producing neurons^41^. In this study, samples that were affected by M-kp10 showed a significant increase in *kiss1ra* gene expression in both hypothalamic and ovarian tissues compared to other groups. The results of the present study represented that M-kp10 has a higher effect on the expression of *kiss1* and *kiss1ra* genes compared to the kp-10 and GnRH3.

*Cyp19a1* gene expression has been reported in both the hypothalamic and ovarian tissues of fish^45^. Increasing the concentration of sex hormones such as E2 has increased the expression of the *cyp19a1* gene in zebrafish^46,47^. The results of a study on European sea bass showed that treatment with sex hormones significantly increases the expression of the *cyp19a1* gene in hypothalamic tissue^48^. Another study showed that the expression of the *cyp19a1*gene in the hypothalamic tissue is low until the vitellogenesis, but in the final stage of oocyte maturation, its level increases significantly^35^. In this study, the expression of the *cyp19a1* gene in both hypothalamic and ovarian tissues of the group exposed to M-kp10 was significantly increased. Oocyte maturation was also high in this treatment; therefore, it seems that high levels of sex hormones and oocyte maturation in M-kp10 treatment are related to increased *cyp19a1* gene expression in the hypothalamic and ovarian tissues.

One study found that injection of the 10 amino acid kisspeptin into European sea bass over 7 weeks increased gonadal growth index and sperm maturation^49^. In female clownfish (*Amphiprion mel-anopus*), injection of human kisspeptin increased vitellogenin synthesis and increased gonadal growth and oocyte growth over 6 weeks^50^. In chub mackerel, the concentration of kisspeptin in the final stages of oocyte maturation increased dramatically, and in *Channa striatus*, injection of mammalian kisspeptin increased the rate of gonadal development^51^. In the present study, groups received M-kp10 and GnRH3 (250 µg/kg) showed a significant increase in the number of oocytes in the final stage of maturation.

## Conclusion

Based on the results of hormone analysis, histology, gene expression in both hypothalamic and ovarian tissues and reproductive indices, Phe6Trp substitution in parallel with N-terminal acetylation resulted in enhancing the reproductive ability of kp-10 significantly. It is suggested that our peptide could be considered as one of the alternative candidates for synthetic hormones in future studies and according to the results it seems that M-kp10 can be used to reproduce other domestic animals like sheep, goats, cattle, horses and pigs.

## Materials and methods

### Fish

The natural spawning season of goldfish takes place in spring and May. Therefore, the fish samples were taken in this season and the samples were in the late stages of sexual maturity. 240 female broodstock fish with an average body weight of 67 ± 5 g were supplied from a fish farm located in the North of Iran (Gilanpoor Artificial Fish Farm, Rasht, Iran). The samples were transferred to the Marine Biology Laboratory at the University of Guilan. After acclimating in a 2000-L aerated fiberglass tank for 7 days, the broodstock was randomly separated into aerated aquaria. The samples were fed twice a day with the feeding powder purchased from Isfahan Mokammel Co. (Isfahan, Iran). The number of samples in each group was 39 (three replications and each replicate: 13 fish per aquarium). The size of the aquariums was 70 × 40 × 40 cm with a volume of 112 L and the photoperiod of experiment was 14L/10D. (There were no exclusions and confounders were not controlled). In total, the number of animals that were used for hormonal and histological analysis and gene expression is shown in Table 1.

**Table 1:** Number of animals used for each protocol.

Protocol	The number of animals
Pre test	90
Blood sampling for hormonal analyses	90
Gene expression and histology	54

### Peptide synthesis

**Figure 8:**
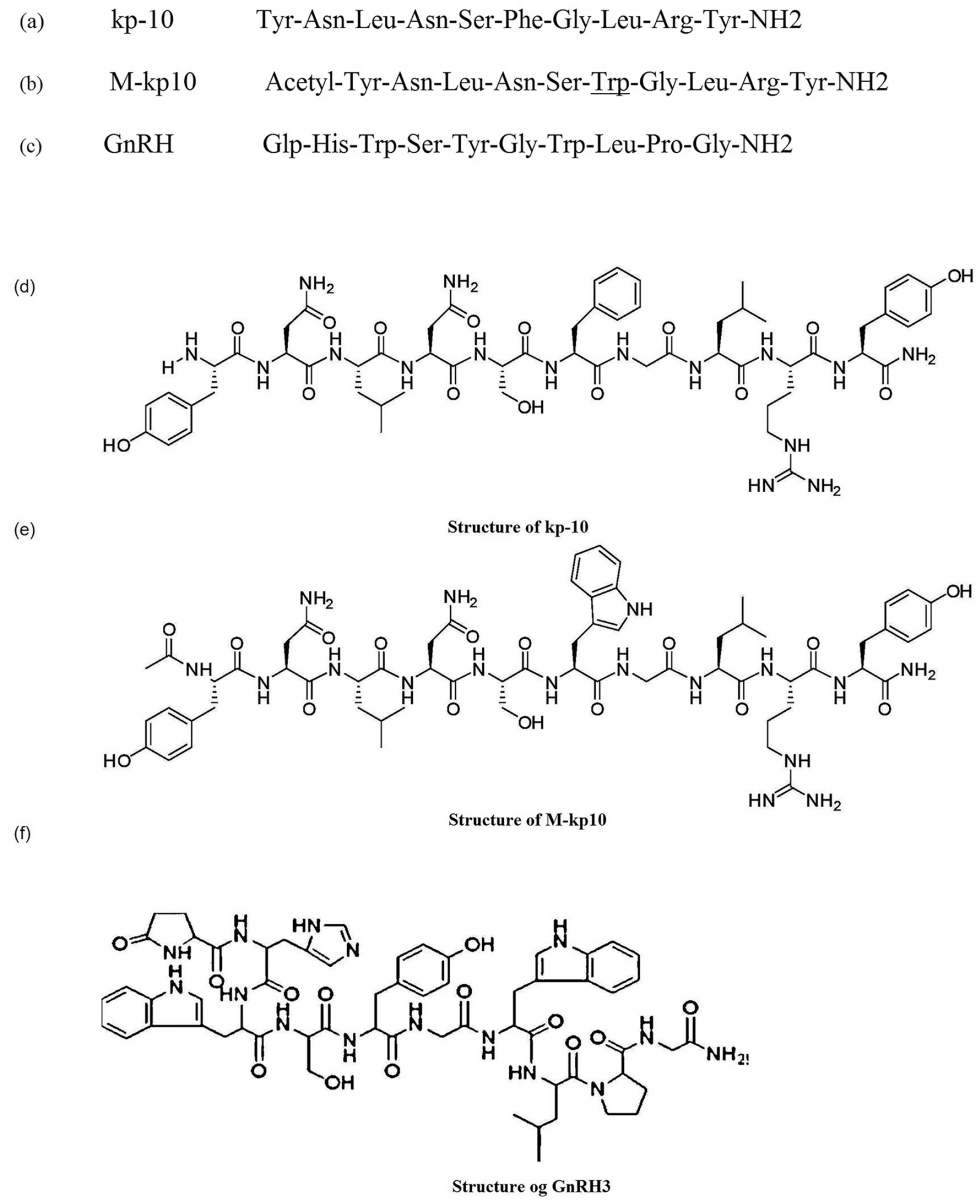
Sequence and structure of peptides. Sequences of (**a**) native kp-10, (**b**) M-kp10 in which Phe 6 is substituted with Trp and the N-terminus is acetylated, and (**c**) GnRH3, and structure of (**d**) kp-10, (**e**) M-kp10, and (**f**) GnRH3.

A novel peptide (M-kp10) was synthesized using site-directed mutagenesis and chemical modification^14,18,29^. Further, kp-10 and GnRH3 were synthesized to compare their biological activities. The peptides were produced based on the sequence of *Carassius auratus* kisspeptin (GenBank accession No. ACI96030.1). Figure 8 displays the sequences of peptides.

The peptides were synthesized using standard Fmoc-solid-phase peptide synthesis chemistry and purified up to 92% (M-kp10), 97% (kp-10), and 94% (GnRH3) by reversed-phase high-performance liquid chromatography (RP-HPLC). Furthermore, electrospray ionization mass spectrometry (ESI-MS) was employed to confirm chemical structures (see supplementary Fig. S1 online).

### Injection and treatments

To inject peptides into fish samples, they should be combined with a dopamine antagonist and a solvent^52^ to help increase physiological efficacy, so in this study, domperidone and propylene glycol were used as a dopamine antagonist and as a solvent, respectively. The injection was performed into the muscle of the pectoral fin in one step.

To obtain the optimal dose of M-kp10, pre-test was first achieved on goldfish. The pre-test experiment was conducted with 5, 20, 50, 100, and 200 μg/kg of M-kp10 along with domperidone. The optimal dose of M-kp10 was determined as 100 μg/kg of body weight of fish. Based on Valipour et al., the optimal dose of kp-10 was 100 μg/kg of body weight^30^.

The female fish were treated in six groups including two controls and four treatments. Treatments included 1- kp-10 (100 µg/kg), 2- M-kp10 (100 µg/kg), 3- GnRH3 with concentration of 100 µg/kg and 4- GnRH3 with concentration of 250 µg/kg. Each peptide was dissolved in propylene glycol (Sigma-Aldrich) with 10 mg of domperidone (Sigma-Aldrich). The controls were 1- domperidone (Domperidone control) contains a solution of domperidone (10 mg/ml) and propylene glycol (95%, V/V) and 2- negative control without any peptide or solutions. The water physicochemical conditions were controlled to the optimum level for the fish (temperature: 21.5 °C, dissolved oxygen: 8.2 ± 0.1 mg/L, pH: 7.2) and monitored daily.

### Ethics statement

This study was carried out in accordance with the recommendations in the ARRIVE Guidelines. Based on the provided recommendation by AVMA guideline for the euthanasia and anesthesia of animals (2020), fish were anesthetized with clove oil (30 mg/l) before blood sampling. For euthanasia, the fish were first anesthetized with benzocaine and then frozen^4^. All experimental protocols were approved by the Ethics Committee in the Faculty of Science, University of Guilan (reference number 2949518).

### Blood and tissue sampling

6 h after the injection of peptides into fish^30^, following anesthesia of the fish, blood sampling was taken (5 samples per each replication) by a needle of a heparinized syringe (2 ml) was inserted into the caudal vein^53^. The blood sample was transferred into tubes and centrifuged at 3000 rpm for 10 min at 4 °C. The separated plasma was stored at −20 °C until hormonal analysis.

24 h post-injection (just before stripping the eggs by hand), the hypothalamic and ovarian tissues were separated from the dissected fish (N = 3) to measure *kiss1*, *cyp19a1*, and *kiss1ra* genes and histological studies of the ovarian growth^54^.

### Hormonal analysis

17β estradiol (E2), follicle stimulating hormone (FSH), luteinizing hormone (LH) were measured using Antibodies ELISA kit (antibodies-online GmbH, Schloss-Rahe-Str. 1,552,072 Aachen, Germany). 17α-20β-Hydroxy-4-peregnen-3-one (17,20β-DHP), 11-keto testosterone (11KT) were measured using Mybiosource ELISA kit (MyBioSource, Inc. P.O. Box 153,308, San Diego, CA 92,195–3308, USA). Cortisol, and lipoprotein lipase (LPL) were measured using Monobind ELISA kit (Monobind ELISA kit, Monobind Inc., Lake Forest, CA 92,630, USA) (see supplementary Table S1, S2, S3 online).

25 μL of the plasma and 50 ml of the Estradiol Biotin reagent (specific monoclonal biotinylated anti-E2 antibody) were added to the wells. After shaking for 30 s, the wells were incubated at room temperature for 30 min. In the next step, 50 μL of estradiol enzyme reagent was pipetted into each well. The mixture was shaken for the 30 s and the wells were incubated for 90 min. The washing buffer (350 μL) and 100 μL of substrate solution were then added to all wells. The wells were incubated again at room temperature for 20 min. The stop solution (50 μL) was lastly added. Finally, the wells were mixed and at the wavelength of 450 nm, the absorbance was read by the ELISA reader (ELISA reader, BioTek, ELx800, Germany). The other hormonal assays and plasma variables were measured according to the related ELISA kit protocol.

### Reproductive indices

24 h after the injection, the ovulated eggs were collected by gently massaging the abdomen of fish^30^ (N = 20 for each group). The eggs were weighed and counted in 1 g of eggs. Further, relative fecundity was calculated as follows^39^.

$$F = N/TW$$

where *F* illustrates relative fecundity, *N* indicates the number of collected eggs, and *TW* demonstrates the total body weight of fish (kg).

For fertilization, milt was added to the eggs in a clean and dry container using the semi-dry method (100 μl of milt per 1 g of eggs). Approximately 4 h after fertilization, 100 eggs were separated from each group and the fertilization percentage was determined by a stereomicroscope (Nikon MSZ 800).

To calculate the hatching percentage, the fertilized eggs were transferred to incubators. After four days, the hatching percentage was computed by the following formula^30^.

$$H = \left( {TL/TE} \right) \times {1}00$$

where *H* denotes hatching percentage and *TE* and *TL* are considered as the total number of collected eggs and larvae respectively.

### Histology

After fixing the ovary in Bouin's solution for 6 h and embedding with paraffin^55^, the tissue blocks were sectioned at 4–5 μm with a rotary microtome (Leica®, Wetzlar, Germany). The tissue sections were fixed on glass slides by albumin and stained with hematoxylin and eosin (H&E). An AmScope digital camera-attached Ceti England microscope was used for photographing slides^56,57^. To count and detect oocytes in each treatment, six replications were considered and 6 slides were prepared for each replicate^58^.

$${\text{Maturation percentage}} = \left( {{\text{mature oocytes}}/{\text{whole oocytes}}} \right) \times {1}00.$$

### RNA isolation and reverse transcription for quantitative RT-PCR (qRT-PCR)

Total RNA was extracted from ovarian and hypothalamic tissues with TRIzol reagent (Invitrogen, USA) according to the manufacturer's recommended protocol. The qRT-PCR was conducted for *kiss1*, *kiss1ra*, and *cyp19a1* as described previously, and the *actb* gene was used as control. The specific primers for *kiss1*, *kiss1ra*, *cyp19a1,* and *actb* were as follows: forward, TGAGTGCAAATCCTCACCGAA and reverse, CAAGATTTAGCCCGACCCAG, forward, TTCCATCAAAGACCCACGAGA and reverse, TTCCACAGAGGCTTGTCCCA, forward, GCCAGCAACTACTACAACAGC and reverse, CCCTGTTCATGCATTCCGAT and forward, GACTTCGAGCAGGAGATGGG and reverse, CCGCAAGATTCCATACCCAGG. Further, the relative expressions of each messenger RNA (mRNA) were analyzed by employing the comparative CT (2 ˆ^-^ΔΔCT) method. The post-PCR melting curve analysis was utilized to monitor the quality of all PCR products. The primer sequences of the intended genes were designed by Oligo Primer Analysis Software 7. The qRT-PCR reactions were set up in 15 μl using template DNA (50 ng), buffer solution (10 × PCR), each primer (2 pmol), dNTPs (0.1 M), Taq polymerase (2U), and double-distilled water (to 15 μl). The thermal cycling conditions were 95 °C for 15 min, followed by 35 cycles of denaturation at 95 °C for 20 s, annealing at 59 °C for 30 s and a final extension at 72 °C for 30 s. The qPCR was conducted using BioFACT™ 2X Real-Time PCR Master Mix (For SYBR Green I, BioFACT, Korea) on a LightCycler® 96 System (Roche, Life Science)^59^. The efficiency of *Kiss1*, *Kiss1ra*, *cyp19a1* and *actb* primers was 94.87, 96.11, 94.91 and 94.11, respectively.

### Statistical analysis

The data were analyzed using SPSS version 19 in Windows 10. The primary values of variables were analyzed initially assuming the normality and homogeneity of variance by Kolmogorov–Smirnov and Levene's test, respectively. The differences between various treatments were evaluated using the one-way analysis of variance (ANOVA) followed by Tukey's post hoc test to identify significant differences among the means of parameters at the confidence level of 95% and all data were expressed as mean ± SEM.

## Supplementary Information

Supplementary Information.

## Data Availability

The data that support the findings of this study are available from the corresponding author upon reasonable request.
