# Supplementary material for: Functional evaluation of a novel kisspeptin analogue on the reproduction of female goldfish
Source: Sci Rep. 2022 Dec 19;12:21944. doi: 10.1038/s41598-022-25950-4 (PMC9763426; doi:10.1038/s41598-022-25950-4)
Supplement: Supplementary file 1 — Supplementary Information. [file 41598_2022_25950_MOESM1_ESM.docx]

**Scientific Reports Journal**

**Functional evaluation of a novel kisspeptin analogue on the reproduction of goldfish**. Hanieh Rabouti, S. Mohsen Asghari, Reihaneh Sariri, Saeed Balalaie, AbdolMajid Valipour, Navid Omidian, Behrooz Heidari. Institute of Biochemistry and Biophysics (IBB), University of Tehran, Tehran, Iran. E-mail: sm.asghari@ut.ac.ir (S.M A)

**Supplementory Fig. S1. HPLC analysis and Mass spectrometry chromatogram of peptides.** HPLC analysis and Mass spectrometry chromatogram of kp-10, M-kp10 and gnrh3 (a) HPLC analysis of kp-10, (b) Mass spectrometry chromatogram of kp-10, (c) HPLC analysis of M-kp10, (d) Mass spectrometry chromatogram of M-kp10, (e) HPLC analysis of gnrh3 and (f) Mass spectrometry chromatogram of gnrh3.

Support information for details of ELISA kits (Supplementary Table S1.), table of Variance Explained (Supplementary Table S2.) and Component Matrix (Supplementary Table S3.) of PCA test is given as tables in the support file.

Supplementary Table S1. Intra assay, inter assay and sensitivity of the ELISA kits.

| Variable | LH | FSH | 17β-E2 | DHP | 11KT | LPL | Cortisol |
| --- | --- | --- | --- | --- | --- | --- | --- |
| Intra assay | CV< 4.8% | CV< 4.8% | CV< 8% | CV< 8% | CV< 8% | CV< 2.78% | CV< 10% |
| Inter assay | CV< 8.2% | CV< 8.2% | CV< 10% | CV< 12% | CV< 10% | CV< 5.20% | CV< 12% |
| Sensitivity | 8.2 pg/ml | 0.1 ng/ml | 25 pg/ml | 0.06 ng/ml | 4.5 pg/ml | 0.44 pg/ml | 91.5 pg/ml |
| Company | Antibodies | Antibodies | Antibodies | Mybiosource | Mybiosource | Monobind | Monobind |
| Assay Type | Sandwich | Sandwich | Competition | Sandwich | Quantitative competitive | Competition | Competition |

Supplementary Table S2. Total Variance Explained.

| Component | Initial Eigenvalues | | | Extraction Sums of Squared Loadings | | | Rotation Sums of Squared Loadings | | |
| --- | --- | --- | --- | --- | --- | --- | --- | --- | --- |
|  | Total | % of Variance | Cumulative % | Total | % of Variance | Cumulative % | Total | % of Variance | Cumulative % |
| 1 | 3.333 | 41.666 | 41.666 | 3.333 | 41.666 | 41.666 | 2.759 | 34.490 | 34.490 |
| 2 | 2.511 | 31.389 | 73.054 | 2.511 | 31.389 | 73.054 | 2.458 | 30.724 | 65.213 |
| 3 | 1.260 | 15.755 | 88.809 | 1.260 | 15.755 | 88.809 | 1.888 | 23.596 | 88.809 |
|  | | | | | | | | | |

Supplementary Table S3. Component Matrix

| Parameters | Component | | |
| --- | --- | --- | --- |
|  | 1 | 2 | 3 |
| Hatching percentage | .949 |  |  |
| Fertilization percentage | .943 |  |  |
| Fecundity | .798 |  | .443 |
| DHP | -.706 |  | .620 |
| LH |  | .898 |  |
| FSH |  | .892 |  |
| E2 |  | .828 | .369 |
| 11KT | -.578 | -.440 | .611 |
